# mTOR与非小细胞肺癌

**DOI:** 10.3779/j.issn.1009-3419.2010.01.13

**Published:** 2010-01-20

**Authors:** 绍发 许

**Affiliations:** 101149 北京 北京市结核病胸部肿瘤研究所 Department of Cellular and Molecular Biology, Beijing Tuberculosis and Thoracic Tumor Research Institute, Beijing 101149, China

肺癌是当今世界上严重威胁人类健康与生命的恶性肿瘤，每年有超过100万人死于肺癌。世界卫生组织报告肺癌和艾滋病将是21世纪危害人类健康最严重的两种疾病。近年来随着分子生物学的发展，磷脂酰肌醇3激酶(phosphatidylinositol 3-kinase, PI3K)/蛋白激酶B(protein kinase B, AKT)/哺乳动物雷帕霉素靶蛋白(mammal target of rapamycin, mTOR)PI3K/AKT/mTOR信号通路的研究逐渐成为热点^[1]^。mTOR信号通路处于细胞生长繁殖、细胞周期调控、生物合成、细胞迁移等调控中心位置，扮演着重要角色^[2]^。mTOR的活化和其下游信号递质的研究为肺癌发生、发展和治疗打开了一个非常有前景的领域。本文将对mTOR与非小细胞肺癌(non-small cell lung cancer, NSCLC)的关系做一综述。

## 1 mTOR的分子结构及功能

雷帕霉素靶蛋白(target of rapamycin, TOR)，是一种非典型的丝氨酸/苏氨酸蛋白激酶，1994年Brown等^[3]^证实并克隆了哺乳动物TOR基因，即mTOR。它是一类进化上非常保守的蛋白激酶，广泛存在于各种生物细胞中。它属于PI3K蛋白激酶类家族，是PI3K信号通路下游的一个效应蛋白，其底物主要控制与细胞生长和增殖密切相关的蛋白质合成。mTOR的分子结构复杂，由2 549个氨基酸残基组成，分子中有数个各自独立而保守的结构域，mTOR分子的氨基端存在20个串联重复序列，每一个重复序列包含2个分别由40个氨基酸残基组成的α螺旋，每个螺旋都有1个亲水基团和1个疏水基团。这种重复结构介导蛋白质之间的相互作用，有利于mTOR定位于浆膜。mTOR分子羧基端的中部是蛋白激酶域，结构和激酶的催化域相似^[4]^。mTOR主要功能是参与膜蛋白转运、蛋白质降解、蛋白激酶C信号和核糖体合成等一系列过程。mTOR被磷酸化激活后，通过调控4EBP和P70S6K两条不同的下游通路，分别控制特定亚组mRNA的翻译，mTOR调控着翻译的生物合成，是蛋白质生物合成的基础^[5]^。

## 2 mTOR与NSCLC的关系

### 2.1 mTOR在NSCLC发生、发展中的作用

mTOR能够加快细胞周期G_1_期-S期的转换，促进细胞增殖。mTOR主要通过两种信号通路调控细胞的生长和增殖：一条是PI3K/AKT/mTOR通路。AKT可直接磷酸化mTOR从而激活它的下游途径，控制着细胞增殖和转化所需特殊蛋白质的翻译，调控细胞生长与增殖。另外一条是AKT/TSC1-TSC2/mTOR通路，TSC是肿瘤抑制因子，*TSC*基因发生突变或缺失时会引起细胞粘附、生长和迁移。哺乳动物细胞中TSC1-TSC2复合物是mTOR的抑制因子，因此在TSC1-TSC2异常的细胞中，mTOR和它下游的效应分子被激活，使得细胞可以不断生长^[6]^。肿瘤发生的主要特点是丧失正常细胞周期调控、细胞过度增殖等。mTOR是G_1_期细胞周期蛋白合成的核心调控者，不仅在正常的细胞生长增殖中起主要作用，而且与正常细胞向癌细胞转化以及癌细胞的生长、增殖密切相关，是细胞生长的中心调控因子。

mTOR信号通路的活化在人类多种肿瘤中均有发生，该通路可以通过多种机制促进肿瘤的发生。这些机制主要包括：①第10染色体丢失的磷酸酶基因(phosphatase and tensinhomologue deleted on chromosome 10, PTEN)被认为是一种重要的肿瘤抑制基因，它与一些肿瘤的发生、发展有密切关系。PTEN的失活可导致PI3K/AKT/mTOR通路活性增加、PI3K突变或者扩增、癌基因受体的活化等^[7-8]^；②PI3K/AKT/mTOR信号通路主要是通过AKT蛋白过度活化和扩增该通路上游的相关受体：c-Met、c-Kit、VEGF、IGF-IR，从而引起该通路的活化^[9-10]^；③血管内皮生长因子(vascular endothelial growth factor, VEGF)是强大的促血管因子，可以刺激内皮细胞的增殖和迁移，并且增加血管通透性，为肿瘤的生长提供必要的氧气和营养物质。VEGF还是体内外血管内皮细胞生长特异的有丝分裂原，通过选择性地与血管内皮细胞膜上高亲和力的受体酪氨酸激酶结合使受体磷酸化，然后进一步激活其下游的相关基因，刺激内皮细胞迁移与增殖；④有研究^[11]^表明：趋化因子受体(chemokine receptor 4, CXCR4)是一种细胞表面受体，它在NSCLC呈过表达。PI3K激酶活化下游AKT、mTOR和P70S6激酶可引起低氧诱导因子-1α(hypoxia inducible factor, HIF-1α)活性增加而引起CXCR4表达增加。HIF-1α是诱导低氧基因和修复细胞氧内环境的核心调节因子，它可引起CXCR4的过表达和趋化行为。PI3K和mTOR抑制剂可抑制HIF-1α的活性和CXCR4的表达，另外诱导野生型PTEN进入NSCLC细胞也可抑制CXCR4的表达。

PI3K/AKT/mTOR通路在NSCLC中呈现活化状态，在NSCLC患者切除的肿瘤组织中，通过免疫组织化学方法可以分析检测到PI3K、AKT、mTOR高水平磷酸化表达。该通路的活化在原发的NSCLC组织中表达，也见于恶变前和恶变的人类支气管上皮细胞，而在正常细胞中基本不表达，可见PI3K/AKT/mTOR通路的活化在从癌前病变向恶性肿瘤的转变中起着重要作用^[12]^。Balsara等^[13-14]^运用免疫组织化学方法检测62例肺癌组织和12例非肺癌组织(4例肺结核和8例炎性假瘤)mTOR蛋白的表达。研究结果显示：mTOR在肺良性疾病组中表达很低，而在NSCLC组织中表达的阳性率为41.7%，明显高于肺良性疾病组，表明mTOR在NSCLC中被激活。mTOR的表达也与NSCLC的pTNM分期有关，随pTNM分期的增加，mTOR的表达也随之增加，提示mTOR的激活与肿瘤进展有关。Tsurutani等^[15]^运用免疫组织化学方法分析NSCLC病人300例、正常组织100例，结果显示：NSCLC病人中大多数PI3K/AKT/mTOR通路活化，而在正常组织中几乎没有(73.4% *vs* 0%; *P* < 0.01)。在300例NSCLC病人中腺癌高于鳞癌(8.1% *vs* 68.5%; *P*=0.04)，各期的病人生存时间均较短，该通路的活化对于Ⅰ期和肿瘤 < 5 cm的早期病人预后较差，提示PI3K/AKT/mTOR通路的活化提示预后不良。Herberger等^[16]^使用免疫染色方法对88例NSCLC病人肿瘤组织进行检测，其中有56例mTOR表达阳性。活化的mTOR和病理类型没有关系。所有mTOR阳性的肿瘤病人的生存期明显短于mTOR阴性的肿瘤病人(死亡危险率=2.57；95%CI：1.35-4.89；*P*=0.004)。多变量*Cox*比例风险回归分析显示，mTOR对于死亡来说是一个独立的预后因素(修正死亡危险率=2.44，95%CI：1.24-4.80；*P*=0.01)。有研究表明尼古丁可以活化PI3K/AKT/mTOR信号通路，另外尼古丁还与肺癌患者对化疗药物的耐受相关。活化过程中可使内源性乙酰胆碱释放，它可作为自分泌或者旁分泌生长因子受体而促进肺癌细胞的发展。PI3K/AKT/mTOR通路的活化同尼古丁诱导细胞相关，尼古丁活化PI3K也可有效地导致Bax(Bcl-2蛋白家族的一员)增加，因此凋亡的功能被抑制。*NNK*(一种烟草特定的癌基因)的发现更支持尼古丁与肺癌的关系^[17, 18]^。以上研究结果表明mTOR在肺癌的发生、发展、转移中可能起到一定作用。

### 2.2 mTOR通路抑制剂与NSCLC的靶治疗

mTOR信号的异常与许多人类疾病有关，因此对该信号系统的研究将有助于这些疾病的靶治疗。由于与肿瘤密切相关，mTOR成为肿瘤治疗的一个重要新靶点。目前研究的比较明确的mTOR抑制剂主要有以下几种：

#### 2.2.1 雷帕霉素(rapamycin)

雷帕霉素是mTOR的特异性抑制剂，其也是最早被发现的mTOR抑制剂，是20世纪70年代从细菌中分离出来的一种大环内酯类抗生素。1997年FDA批准雷帕霉素作为免疫抑制剂应用于肾移植。由于它还具有抗冠状动脉再狭窄的作用，可用于药物洗脱支架。雷帕霉素结合FK506结合蛋白而抑制mTOR，阻止P70S6K和4EBP磷酸化，同时也间接抑制了其它相关蛋白转录和翻译，具有抗菌、免疫抑制和抗肿瘤作用^[19]^。后来，人们发现了雷帕霉素具有抗肿瘤活性。在许多肿瘤组织培养和动物模型中，雷帕霉素均具有浓度依赖性抑制肿瘤细胞生长的活性。实验证明它们可以抑制非小细胞肺癌、前列腺癌、恶性胶质瘤、骨肉瘤、胰腺癌和肾细胞癌中肿瘤细胞的分化并可以诱导其凋亡。雷帕霉素对PTEN失活伴有PI3K/AKT/mTOR信号通路激活的肿瘤效果比较好。野生型PTEN阻止细胞由静止期向增殖期发展，PTEN的失活使得该通路过度活化^[20]^。PTEN的失活大都伴随着PI3K/AKT/mTOR通路被激活，也有报道认为PTEN的失活是PI3K/AKT/mTOR通路激活的原因之一，从而导致mOTR的过度激活，使得下游效应蛋白P70S6K和4EBP过度磷酸化，从而提高了P70S6K和4EBP的活性，而这个过程可被雷帕霉素类药物靶性阻断^[21]^。

#### 2.2.2 Sirolimus

Sirolimus是由Wyeth-Ayest公司研究并开发的新型大环内酯类抗真菌药。与雷帕霉素相比，Sirolimus等雷帕霉素衍生物具有较好的水溶性和更好的稳定性。Sirolimus通过抑制mTOR下游P70S6K和eIF4的磷酸化，从而抑制其蛋白合成，对细胞周期的G_1_期-S期有阻断作用，还能减少cdc-2激酶的活性，促进细胞凋亡。在乳腺、肺、肾等多种人类肿瘤模型中，Sirolimus单独应用或与细胞毒药物联合应用均显示良好的抗癌活性，在体外试验中，特异性抑制剂Sirolimus的抗血管形成作用可以被5-FU加强^[22]^。Neshat等^[23]^发现，对PTEN缺失的肿瘤杂交小鼠使用Sirolimus治疗4周，肿瘤明显缩小，缺失PTEN的小鼠细胞和人类肿瘤细胞系对于Sirolimus的治疗有效，可见Sirolimus对PTEN的缺失、突变的肿瘤细胞作用明显。Margolin等^[24, 25]^关于Sirolimus单药治疗肿瘤的临床可行性研究已经完成，表现出较好的抗肿瘤活性，其药物毒性主要包括消化系统不适、皮肤毒性反应和粘膜炎等。Tsao等^[26]^研究Sirolimus可使对于顺铂耐受的NSCLC病人减少耐受。还可以导致细胞凋亡和减少肿瘤细胞的生长。不幸的是它并不能延长病人的存活率。目前的Sirolimus临床前评估以及Ⅰ期、Ⅱ期临床试验已经完成，都显示出良好的抗肿瘤作用，因而具有更广泛的临床应用前景^[27]^。

#### 2.2.3 其它靶向治疗

Everolimus是新型的口服mTOR抑制剂，在欧美国家用于器官移植后的免疫抑制剂，在肿瘤治疗的早期临床研究中表现出很好的疗效。Everolimus先与FKBP12形成二聚体后再直接与mTOR结合，从而抑制mTOR活性并阻断mTOR信号通路。单药有抗肿瘤活性，且与细胞毒性药物联合具有协同作用，值得在今后进一步开展其单药或与化疗联合治疗肺癌的临床研究^[28]^。早期试验证明，Everolimus不仅对NSCLC有效，同时对小细胞肺癌(small cell lung cancer, SCLC)同样有效^[29]^。其它的mTOR的特异性抑制剂还包括Temsirolimus、Deforolimus等，在临床前期试验中也表现出较好疗效^[30]^。大量的临床试验^[31]^显示，上述mTOR抑制剂虽然不能使肿瘤逆转，但可以抑制肿瘤细胞的增殖，与其它的化疗药物联合应用有望获得更好的治疗效果。

## 3 小结与展望

肺癌靶向治疗经过近十多年来的研究，已取得了许多具有里程碑意义的成绩，但仍存在许多问题困扰肺癌靶向治疗的发展^[32]^。mTOR同p53等其它肿瘤标志物一样，不是NSCLC所特有，在人类多种肿瘤中均有其高表达，所以寻找特定的NSCLC标志物仍是日后人们研究的热点。mTOR作为细胞生长中心协调器的作用已为人们所认识，mTOR信号通路被认为是一个调节细胞周期进程和细胞生长的信号汇聚，mTOR信号通路与细胞的生长、分裂、存活、迁移、自我更新和细胞周期进程等生理过程密切相关。随着分子生物学的研究进展，越来越多的科研单位对mTOR进行了较为深入的研究，但目前仍然还有一些问题亟待解决：比如是否有其它信号通道与PI3K/ AKT/mTOR信号通道存在协同或抑制作用？mTOR抑制剂对NSCLC治疗是否还存在其他机制？随着对mTOR更深入的研究，透彻了解mTOR信号通路和mTOR抑制剂的具体作用机制，不仅能进一步完善细胞信号转导通路，还将有助于解释一些肿瘤的发生机制，并且为肿瘤诊断和治疗提供新的思路。
